# Supplementary figures and images for: Thalidomide promotes degradation of SALL4, a transcription factor implicated in Duane Radial Ray syndrome
Source: eLife. 2018 Aug 1;7:e38430. doi: 10.7554/eLife.38430 (PMC6156078; doi:10.7554/eLife.38430)

Figure 1 - source data 2

A

Related to Figure 1 - figure supplement 2D

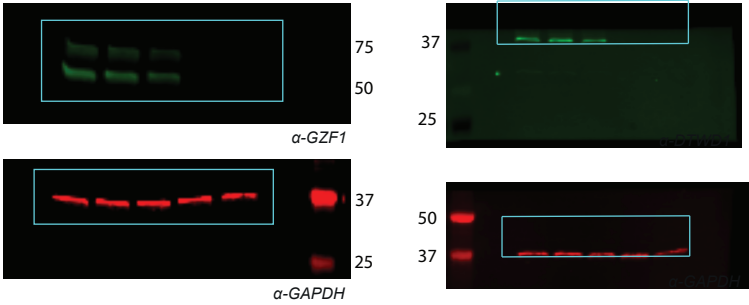

Supplement: Figure 1—source data 15. — Uncropped western blots with the corresponding main or supplementary figure numbers shown. GAPDH loading control is presented with each plot. Size markers (kDa) are indicated. Cyan boxes highlight the cropped segment presented in main or supplementary figures. SALL4 is expressed in two isoforms, which we observe at 150 and 100 kDa apparent molecular weights. Different cell lines appear to express different relative levels of these isoforms. Additional variance in apparent molecular weight may arise from post-translational modifications. [file elife-38430-fig1-data15.pdf]

**Figure 2 - source data 1**

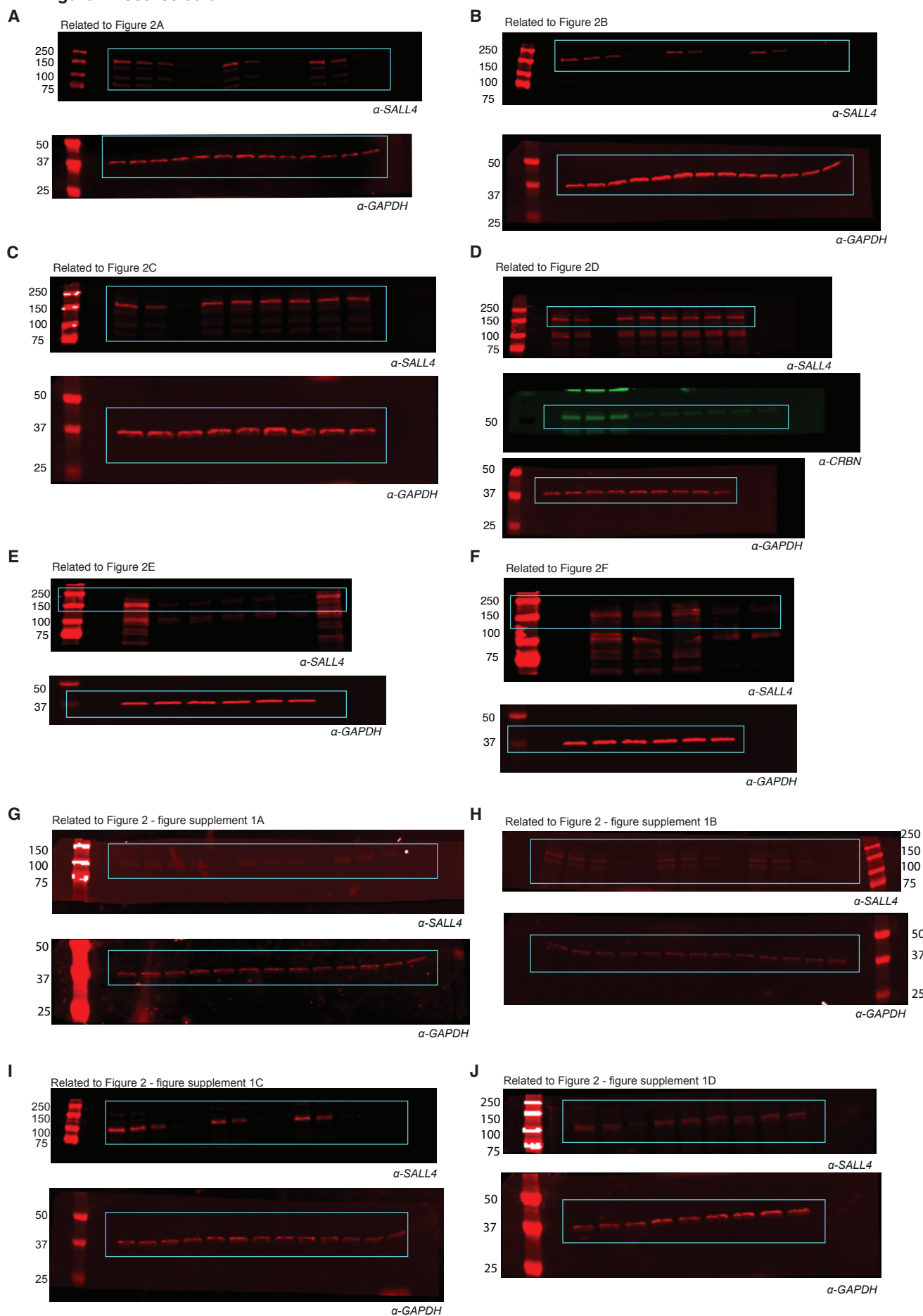

Supplement: Figure 2—source data 1. — (A−J) Uncropped western blots with the corresponding main or supplementary figure numbers shown. GAPDH loading control is presented with each plot. Size markers (kDa) are indicated. Cyan boxes highlight the cropped segment presented in main or supplementary figures. SALL4 is expressed in two isoforms, which we observe at 150 and 100 kDa apparent molecular weights. Different cell lines appear to express different relative levels of these isoforms. Additional variance in apparent molecular weight may arise from post-translational modifications. [file elife-38430-fig2-data1.pdf]

Figure 3 - source data 1

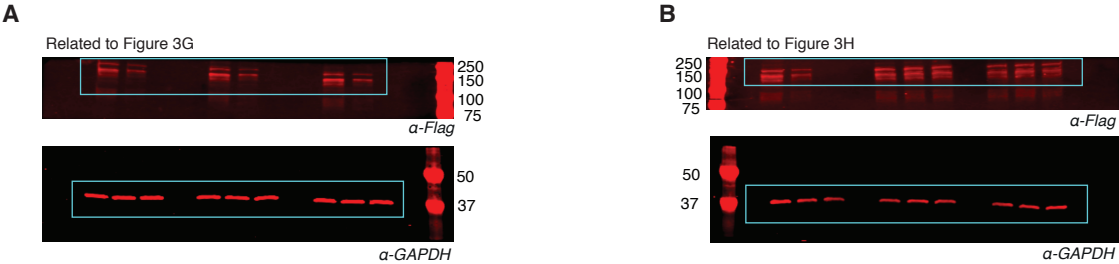

I

J

Supplement: Figure 3—source data 1. — (A−B) Uncropped western blots with the corresponding main or supplementary figure numbers shown. GAPDH loading control is presented with each plot. Size markers (kDa) are indicated. Cyan boxes highlight the cropped segment presented in main or supplementary figures. SALL4 is expressed in two isoforms, which we observe at 150 and 100 kDa apparent molecular weights. Different cell lines appear to express different relative levels of these isoforms. Additional variance in apparent molecular weight may arise from post-translational modifications. [file elife-38430-fig3-data1.pdf]

**Figure 4 - source data 1**

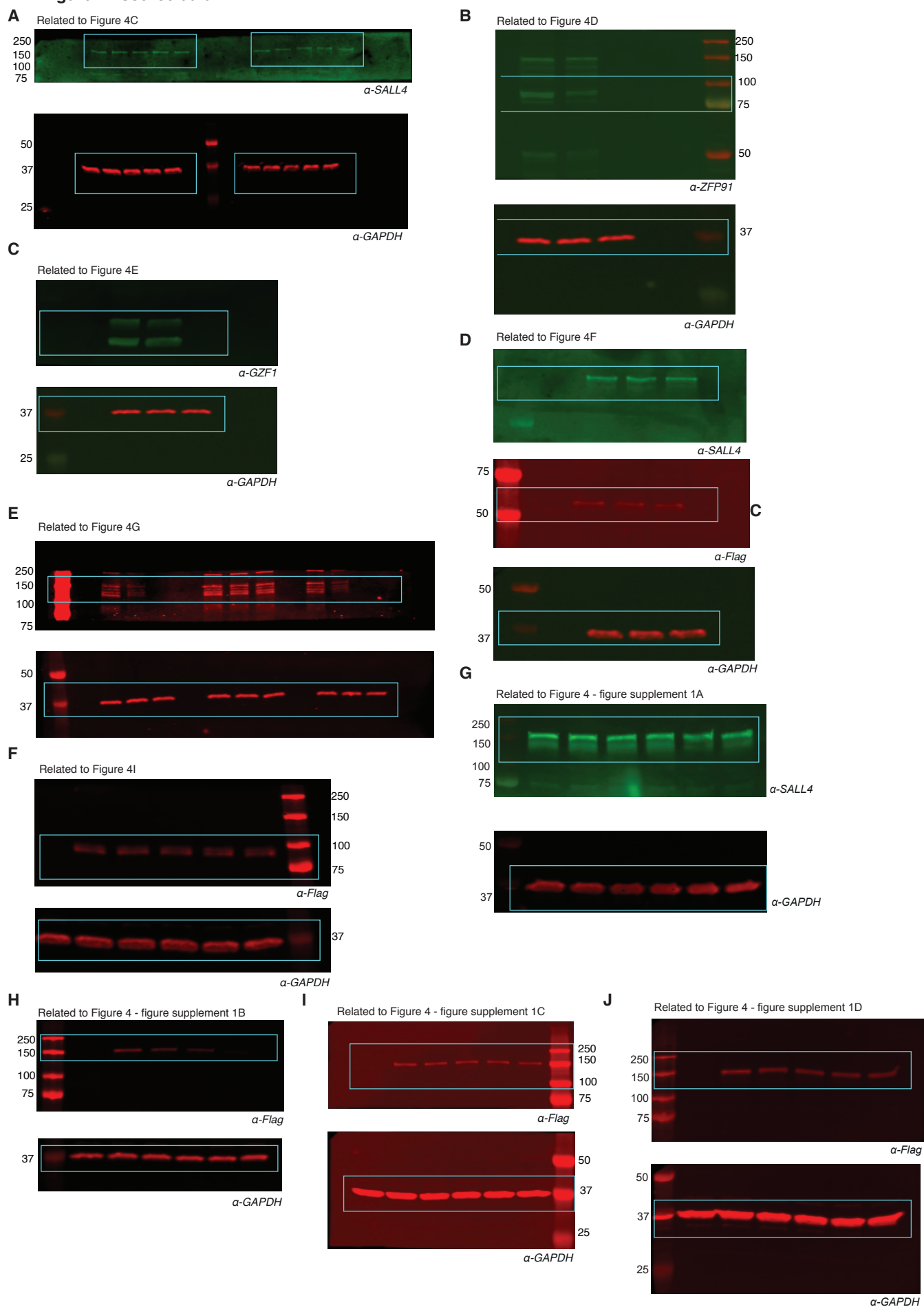

Supplement: Figure 4—source data 1. — (A−J) Uncropped western blots with the corresponding main or supplementary figure numbers shown. GAPDH loading control is presented with each plot. Size markers (kDa) are indicated. Cyan boxes highlight the cropped segment presented in main or supplementary figures. SALL4 is expressed in two isoforms, which we observe at 150 and 100 kDa apparent molecular weights. Different cell lines appear to express different relative levels of these isoforms. Additional variance in apparent molecular weight may arise from post-translational modifications. [file elife-38430-fig4-data1.pdf]
